# Preparation of novel B_4_C nanostructure/Si photodetectors by laser ablation in liquid

**DOI:** 10.1038/s41598-022-20685-8

**Published:** 2022-10-03

**Authors:** Salah S. Hamd, Asmiet Ramizy, Raid A. Ismail

**Affiliations:** 1 Physics Department, College of Science, University of Anbar, Anbar, Iraq; 2 Department of Applied Science, University of Technology, Baghdad, Iraq

**Keywords:** Materials science, Nanoscience and technology

## Abstract

In this study, boron carbide (B_4_C) nanoparticles (NPs) are synthesized by pulsed laser ablation of boron in ethanol at a laser fluence of 6.36 J cm^−2^ pulse^−1^. The effect of numbers of laser pulses on the structural, optical, and electrical properties of B_4_C NPs was studied. X-ray diffraction (XRD) results revealed that all B_4_C nanoparticles synthesized were polycrystalline in nature with a rhombohedral structure. When the laser pulses increased from 500 to 1500, the optical band gap of B_4_C decreased from 2.45 to 2.38 eV. Fluorescence measurements showed the emission of two emission peaks. The Raman spectra of B_4_C nanoparticles exhibit six vibration modes centered at 270, 480, 533, 722, 820, and 1080 cm^−1^. Field emission scanning electron microscope (FESEM) images show the formation of spherical nanoparticles of an average size of 68, 75, and 84 nm for samples prepared at 500, 1000, and 1500 pulses, respectively. The dark I–V characteristics of B_4_C/Si heterojunction photodetectors showed rectification characteristics, and the heterojunction prepared at 500 pulses exhibits the best junction characteristics. The illuminated I–V characteristics of B_4_C/p-Si heterojunction photodetectors exhibited high photosensitivity to white light. The spectral responsivity of the p-B_4_C/p-Si photodetector shows that the maximum responsivity was 0.66 A W^−1^ at 500 nm for a photodetector prepared at 500 pulses. The highest specific detectivity and quantum efficiency were 2.18 × 10^12^ Jones and 1.64 × 10^2^% at 550 nm, respectively, for a heterojunction photodetector fabricated at 500 pulses, The ON/OFF ratio, rise time, and fall time are measured as a function of the number of laser pulses. The photodetector fabricated at 1500 laser pulses showed roughly rise and fall intervals of 1.5 and 0.8 s, respectively.

## Introduction

Boron carbide (B_4_C) is a promising semiconducting material with a rhombohedral lattice space group and a unique set of characteristics that make it a material of choice for a wide range of technical applications. Because of its high melting point and thermal stability, boron carbide is used in refractory applications^1^. At room temperature, the energy gap of 1.7–3.8 eV, a high optical absorption coefficient, and a high electrical resistivity of 10^10^–10^15^ Ω·cm^2^. Boron carbide is the most popular and commercialized boride in the boride family. Because of its high hardness and low mass-density, B_4_C is widely used in industry^3,4^. These properties make boron carbide thin films attractive candidates for applications in the fields of coating materials, electronics, and neutron detectors^5^. Semiconductor nanoparticles (NPs) are very interesting materials because they have unique physical, optical, and electrical properties compared to the bulk material^6^. Various methods were used to prepare boron carbide, such as RF sputtering, pulsed laser deposition, ball milling plasma chemical synthesis, liquid phase reaction, vapor phase reaction, porous alumina template technique, electrostatic pinning, microwave carbo thermal reduction, and metallothermic reduction^7,8^, radio-Frequency Plasma Enhanced Chemical Vapour Deposition (RF-PECVD)^9^. As reported, boron carbide can be found in different morphologies like nanoparticles, nanorods, and nanobelts, depending on the used technique^10^. The physical and chemical properties of boron carbide were found to be dependent on its size and morphology. One of the most promising techniques for the synthesis of colloidal nanomaterials is pulsed laser ablation in liquid (PLAL). In this method, the high intensity of laser pulses can ablates the target in a variety of solvents, including water, and organic solvents. Compared to the above techniques, the PLAL route is easy, compatible, attractive, cost-effective, pure nanomaterial production, preparation of metal oxide nanomaterials, fabrication of nanomaterials with various morphologies, and a green method for the synthesis of metal NPs since no chemically toxic reagents are produced^11^. “To the best of our knowledge”, no data on preparation of B_4_C by laser ablation of carbon target in ethanol has been reported. A fast and efficient route of boron carbide nanoparticles synthesis in ethanol using Nd-YAG laser at various number of laser pulses without using catalyst was demonstrated. XRD, FESEM, UV–Vis spectroscopy, Raman spectroscopy, and photoluminescence spectroscopy were used for comprehensive characterization of colloidal B_4_C NPs. For the first time, a hybrid nanostructured p-B_4_C/p-Si heterojunction photodetector was proposed. The effect of the number of laser pulses on the photodetectors' figures of merit, including current–voltage characteristics, responsivity, specific detectivity, external quantum efficiency, and response time, was investigated and discussed.

## Experimental

**Figure 1:**
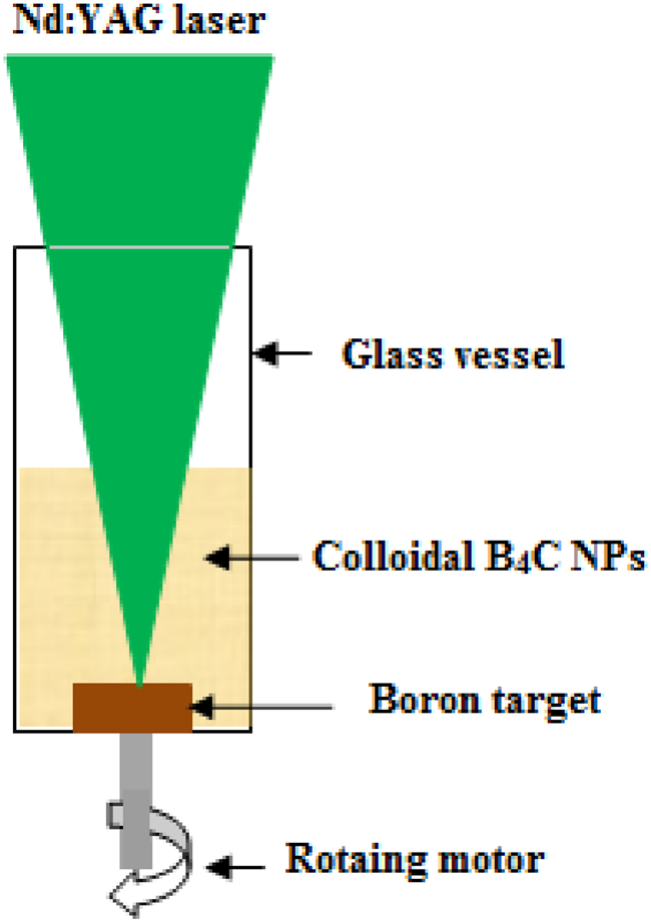
Schematic diagram of PLA system.

**Figure 2:**
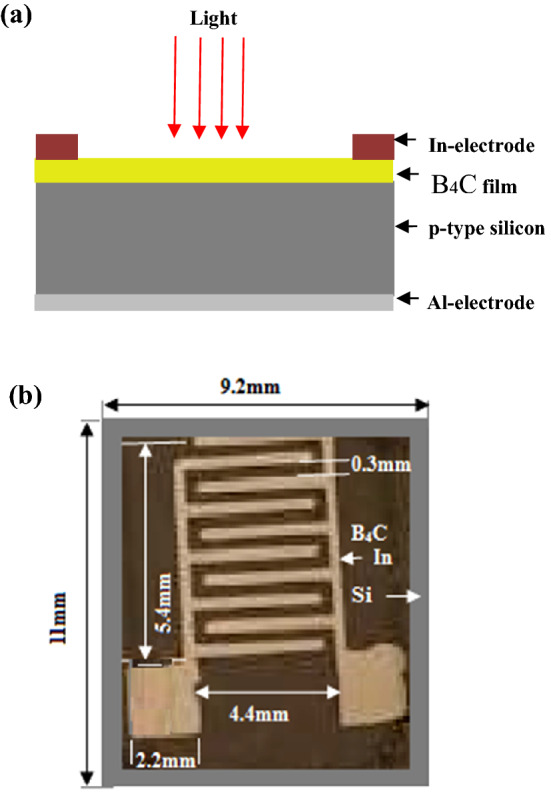
(**a**) Schematic illustration of B_4_C /p-Si heterojunction photodetector and (**b**) optical image of top view of the photodetector with dimensions.

A synthesis of B_4_C NPs was performed by pulsed laser ablation of a high purity boron target in a high purity ethanol solution. Using a 1 cm diameter and 0.5 cm thick hydraulic press, 2 g of high purity (99.9%) boron fine powder (supplied by Sigma-Aldrich) was placed in the pressing mold to prepare boron pellets. The laser beam was focused on a boron target that was placed in a glass vessel with 2 ml of ethanol solution. A focused Q-switched Nd:YAG laser with a wavelength of 532 nm, a pulse duration of 7 ns, and repetition frequency of 6 Hz was used to vertically ablate the boron target at a laser fluence of 6.36 J cm^−2^ pulse^−1^, as shown in Fig. 1. The number of laser pulses used for boron ablation was 500, 1000, and 1500. The laser spot size focused on the boron target was measured by an optical microscope and was around 0.5 mm. The deposition of the boron nanostructure film is carried out using the spin coating method on the silicon and quartz substrates. The structural characteristics were characterized using an X-ray diffractometer (Bruker AXS). The optical absorbance and fluorescence of colloidal B_4_C nanoparticles were measured using a lambda 365 Perkin Elmer UV–VIS spectrophotometer and a (Scinco) FluoroMate FS-2 spectrometer, respectively. The vibration modes of B_4_C NPs were investigated using a Raman spectrometer (532 nm, Preconfigured Raman Spectrometer System). The morphology of the nanoparticles was studied by using FESEM (FEI Verios 460L). The B_4_C/Si heterojunction photodetectors were fabricated by the deposition of B4C film on a 1 cm^2^ polished p-type single crystalline silicon substrate of 350 µm thick using the spin coating method. The electrical resistivity of the (111)-oriented silicon substrate was 3–5 Ω cm. The silicon substrate was cut into square pieces with an area of 1 cm^2^ and then cleaned using RCA technique. Ohmic connections were made on B_4_C and the back side of Si by depositing In and Al films, respectively, as illustrated in Fig. 2a. The optical image of top view of photodetector with dimensions is shown in Fig. 2b, the effective photosensitive area of the photodetector was around 91 mm^2^. At room temperature, the current–voltage characteristics of B_4_C/p-Si were measured with a digital DC power supply, an electrometer, and a lamp source. The light intensity was measured using a silicon power meter. A calibrated Jobin–Yvon monochromator was used in order to determine the photodetector's responsivity, detectivity, and quantum efficiency under bias condition.

## Results and discussion

**Figure 3:**
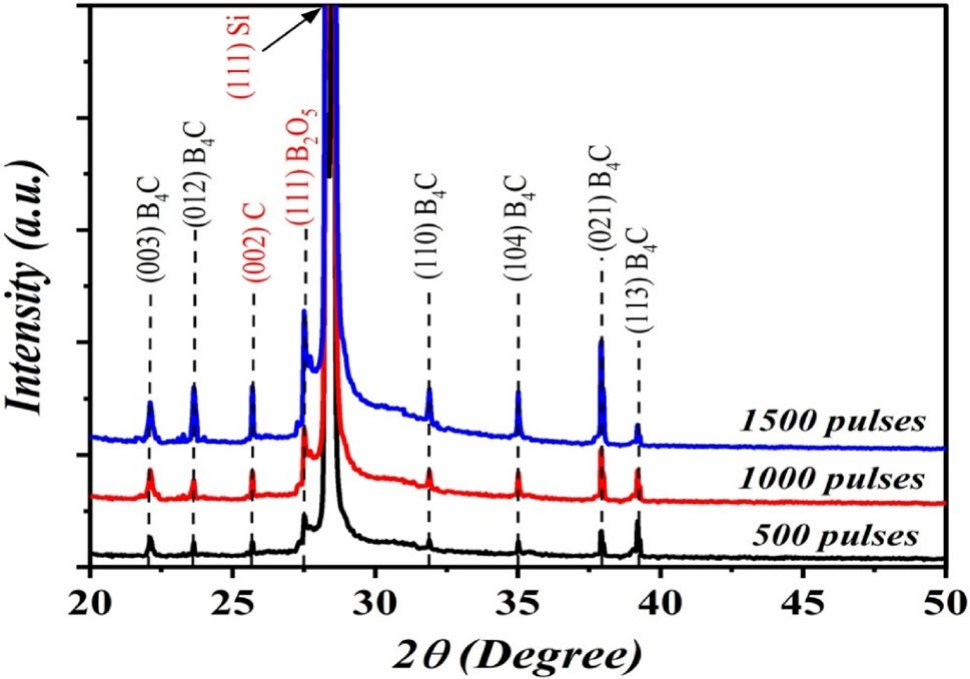
XRD patterns of B_4_C NPs prepared at various number of laser pulse.

Figure 3 illustrates the XRD pattern of B_4_C NPs synthesized at various numbers of pulses and laser fluence of 6.36 J cm^−2^ pulse^−1^. The XRD pattern of B_4_C synthesized at 500 laser pulses showed the existence of eight peaks at 2θ = 22°, 23.6°, 25.6°, 27.5°, 31.8°, 35°, 37.9°, and 39.2° corresponded to (003), (012), (002), (111), (110), (104), (021), and (113), planes, respectively. All of the observed XRD peaks are indexed to polycrystalline rhombohedral B_4_C according to JCPDs #35-0798, and one peak belongs to carbon according to JCPDs #41-189^12^. As clearly seen in Fig. 3, the intensity of reflection peaks increased as laser pulses increased, which indicated that the crystallinity of B_4_C NPs was improved as laser pulses increased. A small shift in 2θ of the reflection peaks was observed when the number of pulses increased from 1000 to 1500, indicating the presence of structural defects arising from stress.

The average crystallite size (D_ave_) of B_4_C synthesized at different number of laser pulses was calculated using Scherrer's equation:

1 $${D}_{ave}=\frac{0.9\lambda }{\beta cos\theta}$$

where λ is the wavelength of X-ray of Cukα source and the β is the full width at the half maximum in units of radians. The average crystallite size of B_4_C NPs prepared at 500, 1000, and 1500 laser pulses was calculated for (021) plane and found to be 50.7, 59.1, and 70.9 nm, respectively. The dislocation density ($\delta$) and strain ($\varepsilon$) that accompanied the laser ablation process were estimated using the following equations:

2 $$\delta ={1/D}^{2}$$

3 $$\varepsilon =\frac{\beta \cdot coscos \theta }{4}$$

Table 1 illustrates the crystallite size, lattice constants, dislocation density, and strain of B_4_C nanostructure films at various laser pulses. The lattice constant (a) and (c) of B4C nanoparticles as a function of laser pulses were determined from XRD patterns and by using rhombohedral unit cell equation as shown in Table 1

**Table 1:** Crystallite size, strain, dislocation density and lattice constants of B4C NPs along (021) plane.

No. laser pulses	FWHM (deg)	D (nm)	δ (nm)^−2^* 10^–4^	ε *10^–5^	a(Å)	b (Å)	c (Å)
500	0.5951	50.7	38	68	4.47	3.5	12.05
1000	0.5038	59.1	28	58	5	3.40	12.02
1500	0.4334	70.9	19	48	4.76	3.41	12.05

4 $$\frac{1}{d^{2}}=\frac{{h}^{2}}{{a}^{2}}+\frac{k}{b^{2}}+\frac{l^{2}}{c^{2}}$$

**Figure 4:**
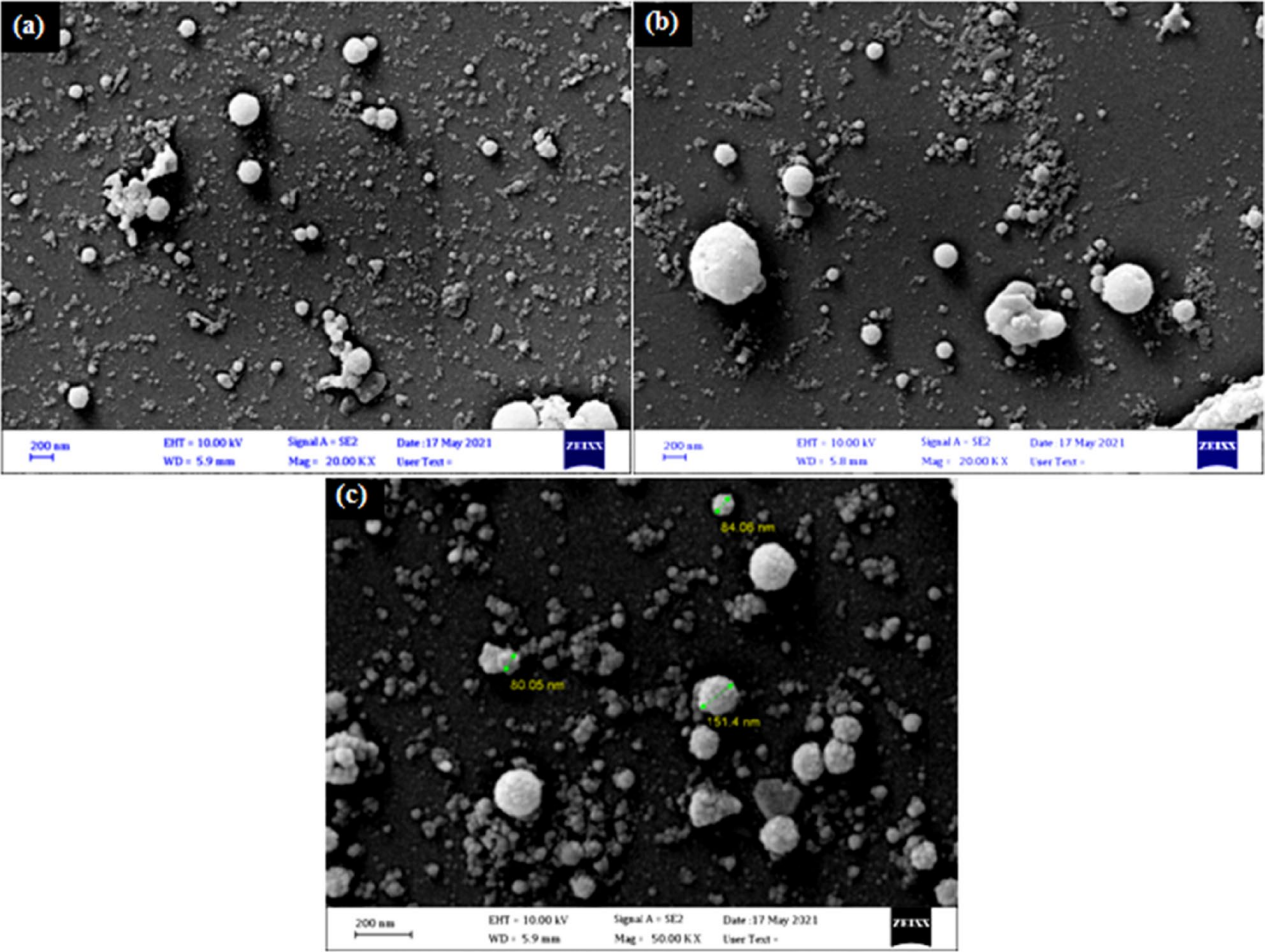
FESEM images of B_4_C NPs synthesized at different laser pulses prepared at (**a**) 500, (**b**) 1000, and (**c**) 1500 pulses.

**Figure 5:**
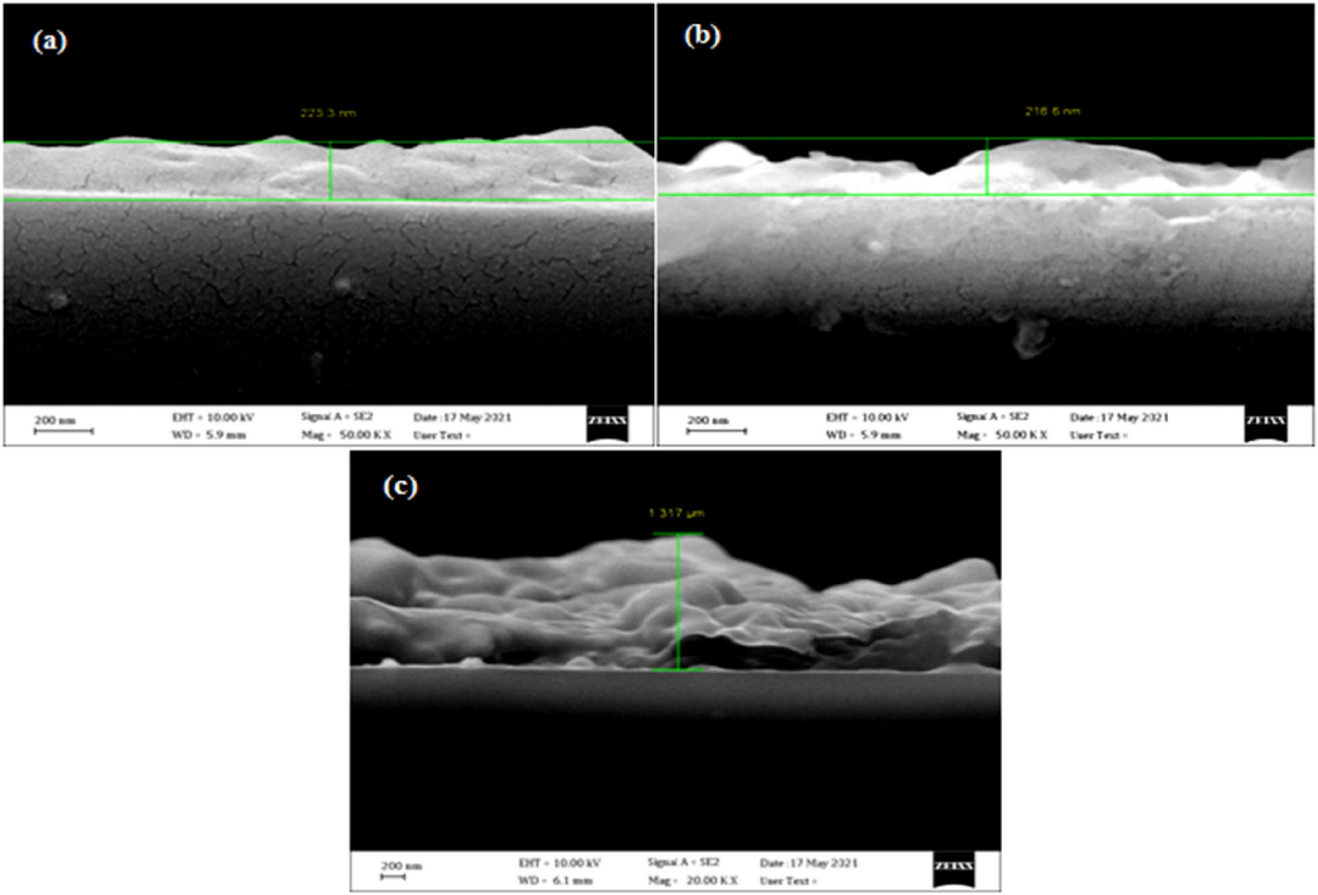
FESEM images of cross-section images of B_4_C NPs synthesized at different laser pulses prepared at (**a**) 500, (**b**) 1000, and (**c**) 1500 pulses.

Figure 4 illustrates the FESEM images of B_4_C NPs prepared at various numbers of laser pulses. In Fig. 4a, the FESEM image shows the formation of spherical particles of different sizes. The average particle size was 35 nm, and some agglomerated particles were observed. The existence of clusters of various sizes was observed, which can be attributed to the non-uniform heat distribution caused by the TEM_00_ laser intensity distribution, which resulted in the ejection of B_4_C NPs via various ablation mechanisms. Because of the increased ablated volume, the particle concentration in the liquid increased as a result of increasing the number of laser pulses. Increasing the laser pulses causes the production of small B_4_C droplets, which are fractured by their interaction with the incoming laser beam, followed by quick quenching, resulting in the formation of bigger nanoparticles, as shown in Fig. 4b. Increased laser pulses (Fig. 4c) result in the formation of bigger spherical nanoparticles as well as increased agglomeration and aggregation of B_4_C NPs as well as platelet morphology with different sizes. The influence of laser pulses on the morphology of B_4_C NPs can be described by the plasma properties formed on the surface target. The high free surface energy of the nanoparticles as the attractive “Van der Waals potential” of the laser pulses grows, so does the density of agglomerated and aggregated particles. The cross section images show that the thickness of the B_4_C nanostructure films was 78, 216 nm, and 1.3 µm corresponding to the 500, 1000, and 1500 laser pulses, respectively. Figure 5 shows the cross-section images of B_4_C nanostructure films prepared at various number of laser pulses. It is clear that the films have rough surfaces, with the presence of hills.

**Figure 6:**
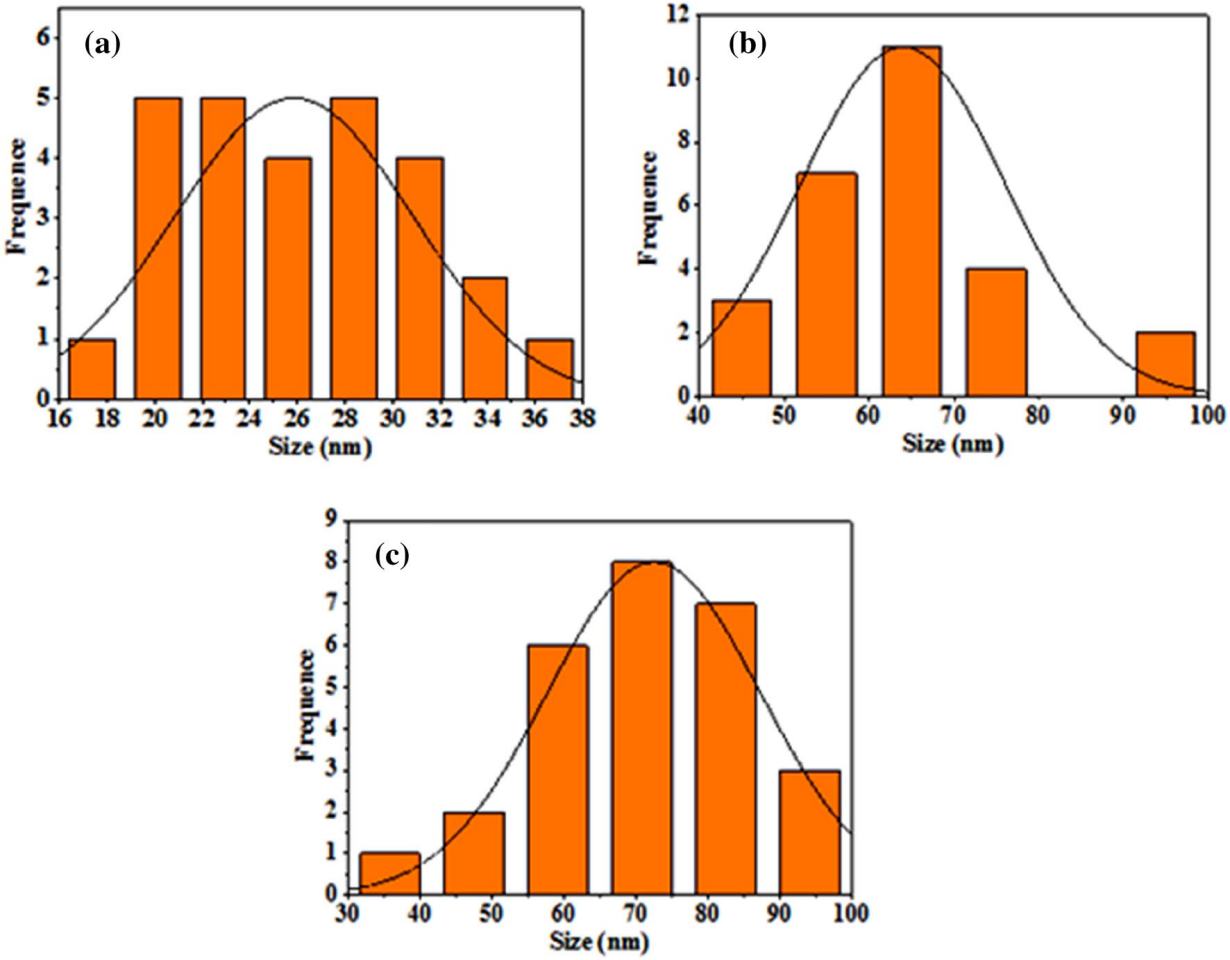
PSD of B4C NPs prepared with (**a**) 500, (**b**) 1000, and (**c**) 1500 pulses.

Figure 6 illustrates the particle size distribution PSD of B_4_C nanoparticles produced with varying numbers of laser pulses. The PSD was determined from FESEM images with aid of image J software. The sample prepared at 500 pulses have PSD extended from 17 to 38 nm with average size of 26 nm. Increasing the number of laser pulses to 1000 causes particle size to increase and the PSD to widen, since a PSD varying from 40 to 100 nm with average particle size of 65 nm. The sample was prepared with 1500 pulses exhibits nearly Gaussian distribution with average size of 70 nm.

**Figure 7:**
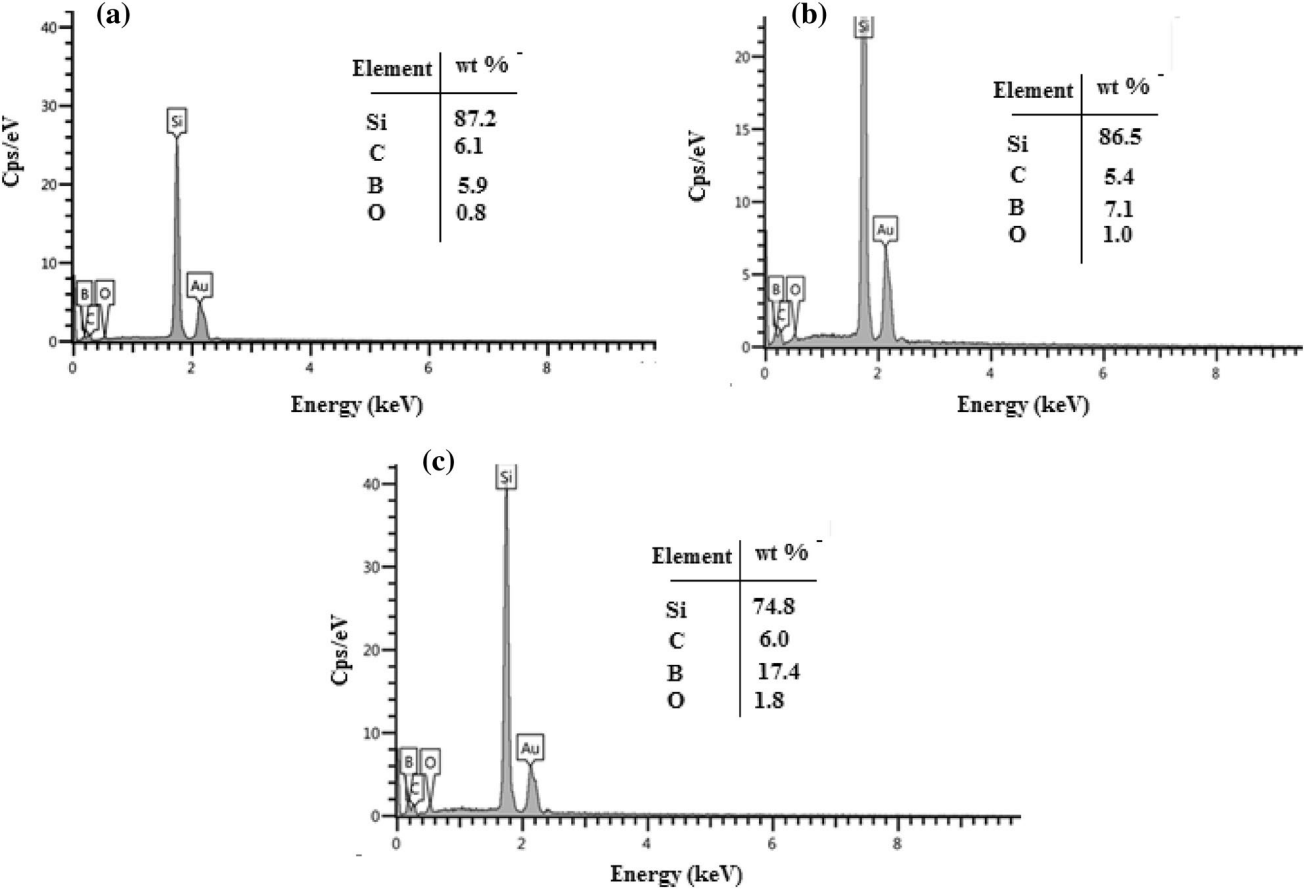
EDX spectra of of B_4_C NPs synthesized at different laser pulses prepared at (**a**) 500, (**b**) 1000 and (**c**) 1500 pulses.

The effect of the number of laser pulses on the EDX spectra of B_4_C nanostructure films deposited on the silicon substrate is given in Fig. 7. These figures confirm the presence of boron, carbon and oxygen elements, and silicon as a substrate. No other elements were detected, which indicates the purity of the product. The [B]/[C] weight ratio determined by EDX analysis was used to estimate the product's stoichiometry, as shown in the inset of Fig. 7. The ratio of stoichiometric B_4_C is 4, while the B_4_C NPs prepared at 500, 1000, and 1500 laser pulses were 1,1.3, and 2.9, respectively. The samples prepared at 500 and 1000 are off-stoichiometric, while the sample prepared at 1500 pulses is near-stoichiometric.

**Figure 8:**
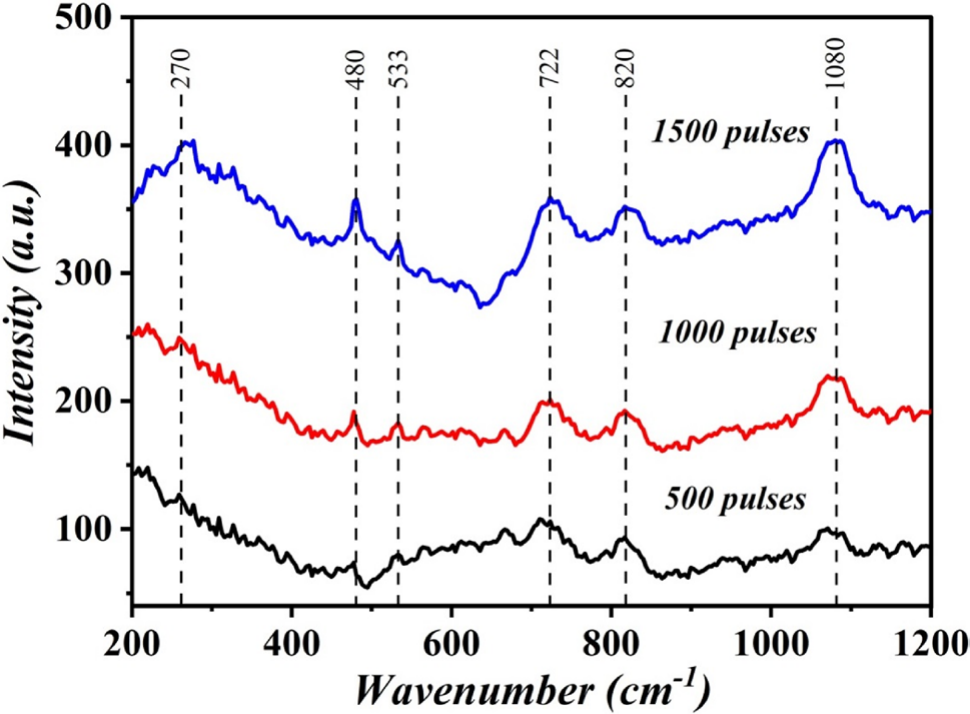
Raman spectra of B_4_C NPs synthesized at different laser pulses.

Figure 8 presents the influence of the number of laser pulses on the Raman spectra of B_4_C NPs. The resulting spectra are characterized by a series of peaks ranging from 200 to 1200 cm^1^ that are ascribed to crystalline boron carbide^13,14^. Six vibration modes were detected in the Raman spectra of B_4_C NPs; all of these bands are B_4_C-related. The peak at 270 cm^−1^ is related to disorder-activated acoustic phonons^1,7^. The strong peak at 480 cm^−1^ is attributed to C-B-C chain stretching, the peak at 533 cm^−1^ is the E_g_ mode of icosahedral boron, the fourth peak centered at 722 cm^−1^ belongs to the E_g_ mode of icosahedral, The fifth broad peak at 820 cm^−1^ is from the A1g mode of icosahedral boron, and the sixth broad peak at 1080 cm^−1^ is associated with icosahdron breathing^15^. The considerable increase in Raman band intensity with increased laser pulses may be due to the increasing B_4_C NP concentration.

**Figure 9:**
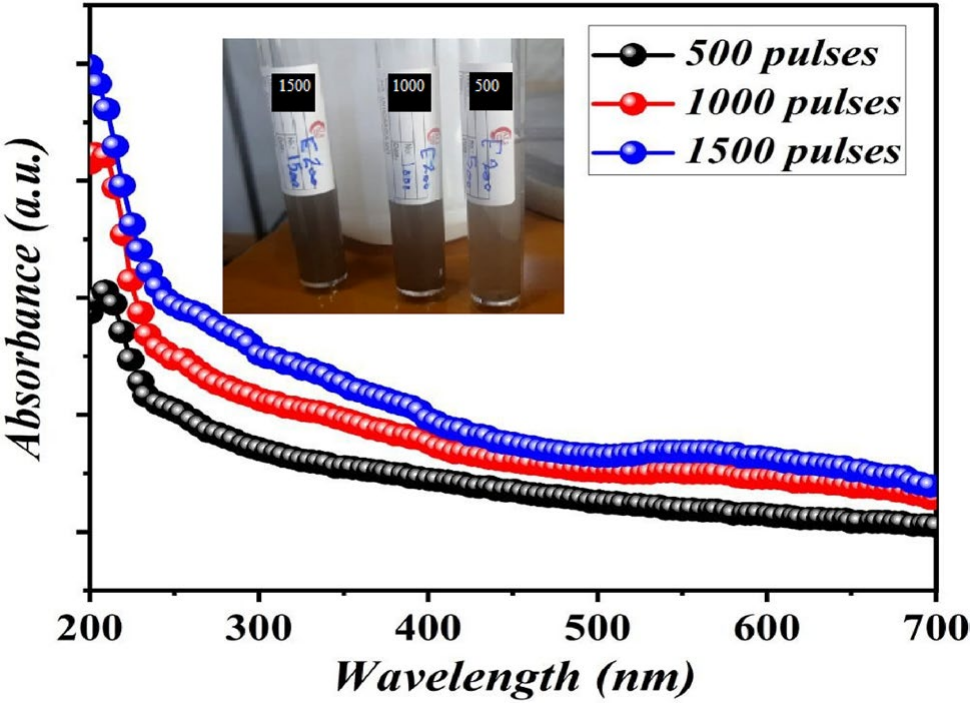
Optical absorption of B_4_C colloid prepared at different number of laser pulses. Inset is the photograph of B4C colloid prepared at different number of laser pulses.

The optical properties of colloidal B_4_C NPs produced with different laser pulses were studied. The effect of laser pulses on the optical absorbance of B_4_C NPs is illustrated in Fig. 9. Because the concentration of nanoparticles and particle size increased with the higher number of laser pulses, absorption also increased slightly. Decreasing the absorption with wavelength indicates the semiconducting nature of the boron carbide. The inset of Fig. 8 illustrates an image of B_4_C colloid nanoparticles generated using a number of laser pulses. As clearly seen, the color of colloidal B_4_C NPs changes from light brown to dark brown as laser pulses increase due to an increase in ablated nanoparticle concentration.

**Figure 10:**
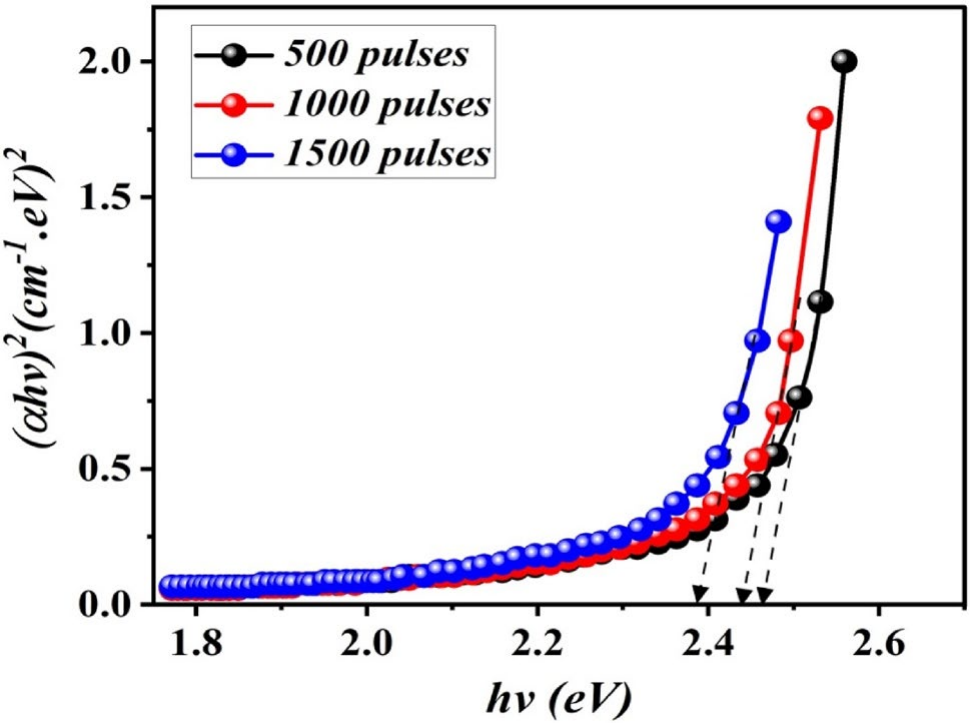
Variation of (αhѵ)^2^ against photon energy (hѵ) of B_4_C colloid prepared at different number of laser pulses.

The optical energy band gap was calculated using the Tauc plot according to the following equation:

5 $${\left(\alpha h\nu \right)}^{2}=A\left(h\nu -{E}_{g}\right)$$

were h is the Planck’s constant, α is the absorption coefficient, ν is the frequency, A is the parameter of edge width, and n is the exponent that determines the transition type^16,17^. Figure 10 illustrates the variation of (αhѵ)^2^ with photon energy (hѵ) and the energy gap can be calculated by extrapolating the linear portion to the photon energy axis^18^. The obtained results verified that as the number of laser pulses increased from 500 to 1500, the optical energy gap of B_4_C reduced from 2.45 to 2.38 eV. This variation in band gap values as particle size increases, which is consistent with SEM investigation. The value of the obtained energy gap is larger than that of bulk boron carbide (2.09 eV) due to the quantum size effect^19^. We believe that increasing the laser pulses leads to reducing the energy gap. Furthermore, we think that the stoichiometry of B_4_C NPs affects their energy gap^20^.

**Figure 11:**
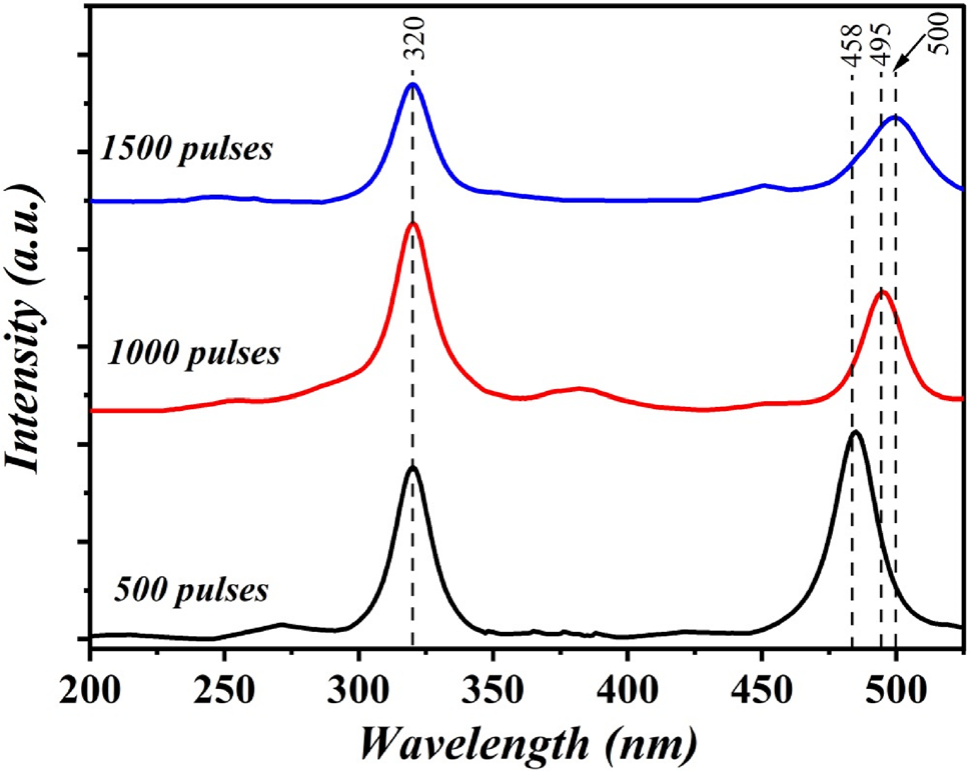
Fluorescence spectra of B_4_C NPs prepared at different number of laser pulses.

Figure 11 shows the fluorescence spectra of B_4_C nanostructure films synthesized at various numbers of laser pulses and fitted with a Gaussian function when excited with a laser beam having a wavelength of 320 nm. The sample prepared at 500 pulses exhibits an emission peak centered at 485 nm, which corresponds to an energy of 2.55 eV. This is related to electron–hole pair radiative recombination at extrinsic impurity centers. The fluorescence of the B_4_C film prepared with 1000 pulses showed a band at 495 nm that corresponded to an energy of 2.5 eV. The fluorescence spectrum of B_4_C film synthesized with 1500 pulses showed an emission band located at 500 nm, which corresponded to an energy of 2.48 eV, which related to band-band recombination of free electrons. The maximum fluorescence intensity was found for B_4_C film prepared at 1500 pulses due to the high concentration of nanoparticles. As reported, the emission peak of bulk B_4_C was centered at 793 nm^21^. This blue shift is due to the effect of quantum confinement^22^. The obtained values are well matched with the energy gap values obtained from optical absorption. The obtained data showed good agreement with reported data^23,24^.

The positive Hall coefficient of the B_4_C nanostructure films confirmed that they are p-type, which agrees with the reported data^25^. As shown in Table 2, the electrical conductivity and mobility of B_4_C nanostructure film decreased as the laser pulses increased as a result of the large concentration of trapping centers and surface states. The charge transfer in B_4_C is caused by hopping processes that occur within these partially filled gap states, which are increased by a contribution from free holes. On the other hand, when the number of laser pulses increases, the conductivity decreases due to the formation of a boron-rich film.

**Table 2:** Effect of number of laser pulses on electrical conductivity and mobility of B_4_C film.

No. laser pulses	Conductivity (o.cm)^−1^	Mobility(cm^2^V^−1^ s^−1^)	Type
500	2.07	33	p
1000	1.84	11.6	p
1500	1.75	4.9	p

**Figure 12:**
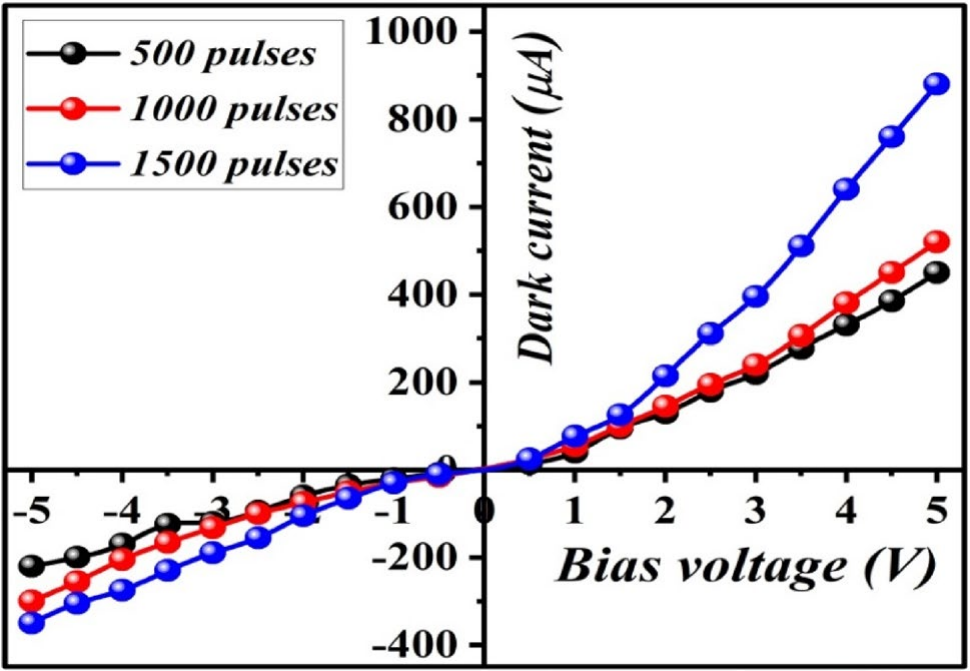
Dark I–V characteristic of isotype B_4_C/Si heterojunction under forward and reverse bias prepared at different number of laser pulses.

**Figure 13:**
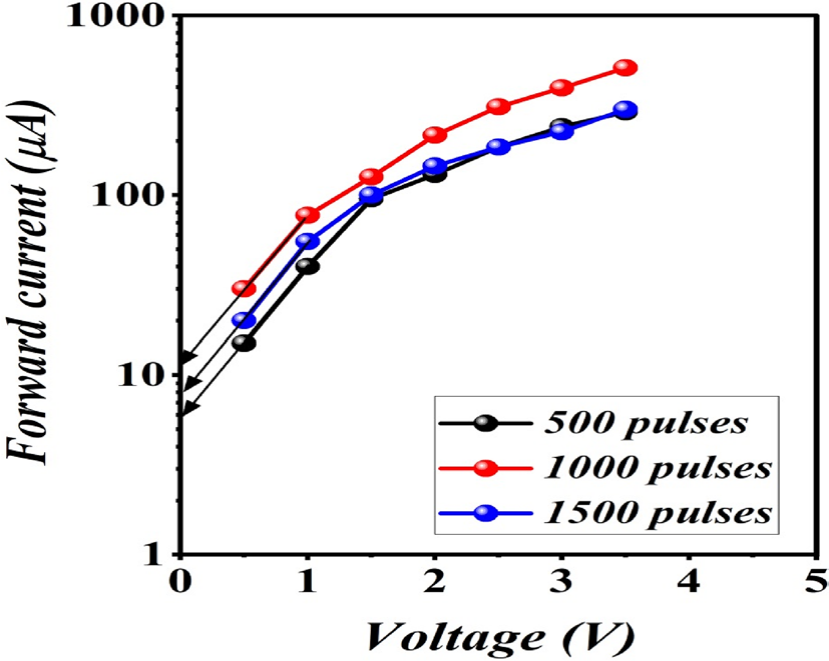
Semi-logarithmic I_f_–V plot of heterojunctions.

Figure 12 depicts the dark current–voltage characteristics curves of isotype p-B_4_C/p-Si heterojunctions fabricated with different numbers of laser pulses measured at room temperature at bias voltage ranging from + 5 to − 5 V. The current transport mechanism of these heterojunctions is governed by the double-Schottky-diode model. The interface state acting as a donor causes the negative space charge in the two depletion regions. The forward current increased slightly with bias up to 1 V and then increased exponentially, indicating that the diffusion current is dominant. The reveres current was found to increase with voltage due to an increase in surface leakage passing current through the heterojunction's edges^26^. Actually, the I–V characteristics of isotype B_4_C/Si are considered as two Schottky diodes connected back-to-back. From semi-logarithmic I_f_–V plots in the forward direction, the saturation current density of the heterojunctions prepared with 500, 1000, and 1500 pulses were determined and found to be 7.5, 15, and 9 μA cm^−2^, respectively, as illustrated in Fig. 13. The ideality factor (n) of the heterojunctions was calculated using the following diode equation:

6 $$n=\frac{{k}_{B}T}{q}\frac{\Delta V}{ln\frac{\Delta I}{{I}_{s}}}$$

where q represents the electron charge, k_B_ is the Boltzmann constant, T is the operating temperature, and Is represents the saturation current. By using Eq. (3), the ideality factor was estimated and found to be 6.5, 9.4, and 8.7 for heterojunctions fabricated with 500, 1000, and 1500 pulses, respectively, indicating that the heterojunction prepared at 1000 pulses provides the best junction properties. For high performance silicon photodiodes, the ideality factor is slightly greater than unity.

A large ideality factor (n ≫ 1) value indicates that the B_4_C–Si junction has poor characteristics due to the surface traps, interface charge, inhomogeneities, series resistance, an structural defects^27,28^. We also attributed the high ideality factor value to a large mismatch in lattice constants between the B_4_C and Si substrates. The mismatch of lattice between B_4_C and the silicon substrate was calculated from XRD analysis and was found to be 9%, which affects the junction characteristics.

**Figure 14:**
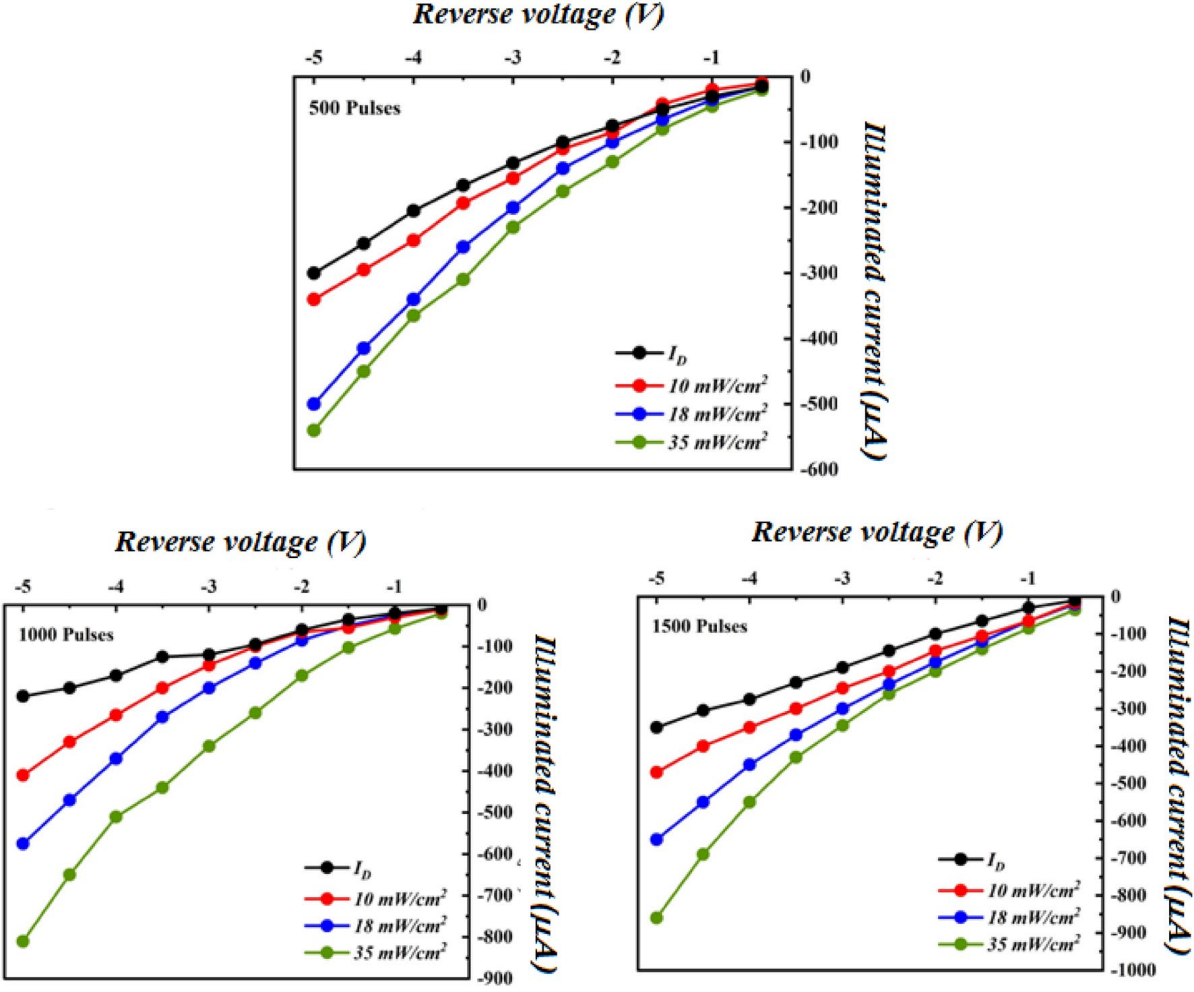
Dark and illuminated I–V characteristic of B_4_C/Si heterojunction photodetectors at various white light intensities synthesized with different number of laser pulses.

The effect of number of laser pulses on the illuminated I–V characteristics of of B_4_C/Si heterojunction photodetectors under various light intensities is shown in Fig. 14. After illumination, the photodetector's current increased as a result of generation of many e–h pairs in the depletion region and diffusion length. No saturation was observed in the photocurrent indicates the photodetectors have linearity characteristics. The linear dynamic region (LDR) of the photodetectors, which represents the measure of linearity, was calculated from a plot of photocurrent versus light power and found to be 40.6, 11.3, and 7.78 at light intensity of 35 mW cm^−2^ for photodetectors fabricated at 500, 1000, and 1500 pulses, respectively. On the other hand, as shown in Fig. 14, the expansion of the depletion region causes an increase in photocurrent with bias voltage.

As illustrated, the photodetector's photocurrent increases as light intensity increases, leading to the generation of a high number of e–h pairs^29,30^. The photocurrent of B_4_C/Si HJ prepared at 1500 was larger than that of the other HJs due to the larger grain size and large mobility of B_4_C film, which increases the diffusion length of minority carriers according to the following equation:

$${L}_{e}=\sqrt{\frac{kT}{e}\mu }\tau $$

where L_e_ is the diffusion length of electron, k is Boltzmann constant, e is the electron charge, µ is the mobility, and τ is the minority carrier lifetime.

The spectral responsivity define as the of photocurrent I_ph_ to the light power P_λ_ ratio at certain wavelength as shown in the following equation:

$${R}_{\lambda }=\frac{{I}_{ph}}{{P}_{\lambda }} $$

**Figure 15:**
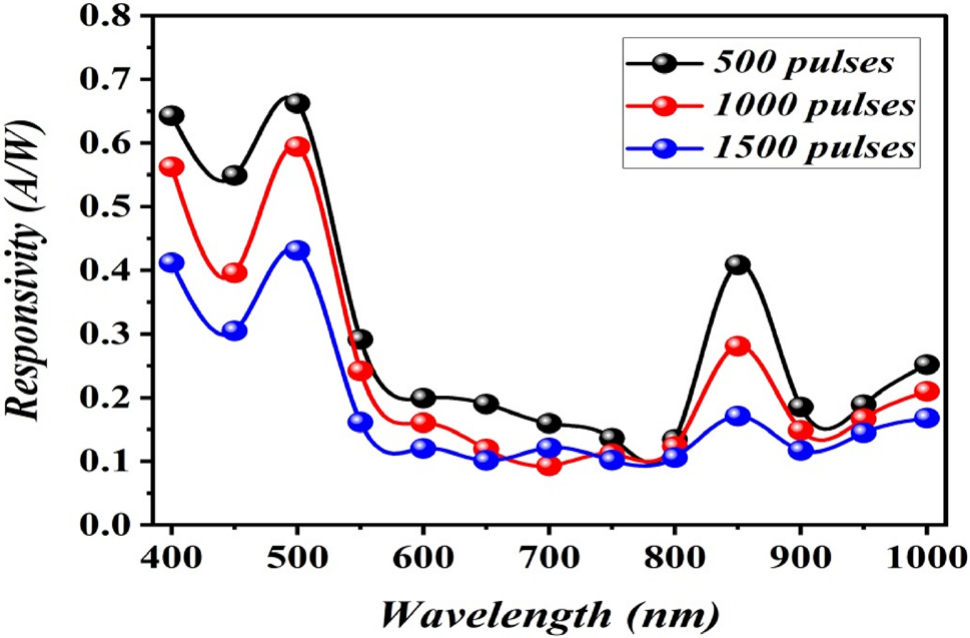
Spectral responsivity plot of B_4_C/p-Si photodetectors fabricated at different number of laser pulses.

This parameter is consider is one the most significant figures of merit of the silicon photodetectors^25^. Figure 15 shows the spectral responsivity of B_4_C/p-Si HJs photodetectors measured at a bias voltage of − 5 V prepared at different number of laser pulses. It is clear that all the fabricated photodetectors have two response peaks. As illustrated in Fig. 15, the located of the peak position and the responsivity value are extremely dependent on the number of laser pulses. All the photodetectors operated in the spectral range of 400–1000 nm, since the photons at short wavelengths are absorbed in the B_4_C film and increase the responsivity in the range of 400–520 nm. While the photons with wavelengths longer than the absorption edge of B_4_C film (λ_c_ = 520 nm) are transmitted into the silicon substrate and increase the responsivity in the range of 520–1000 nm, no detection was observed for wavelengths > 1000 nm due to the absorption edge of the silicon substrate (λ_c_ = 1100 nm). The photodetector fabricated with 500 pulses exhibits two peaks response, the first one at 500 nm with a responsivity of 0.66 A W^−1^ and the second peak located at 850 nm with a responsivity of 0.4 A W^−1^.

**Figure 16:**
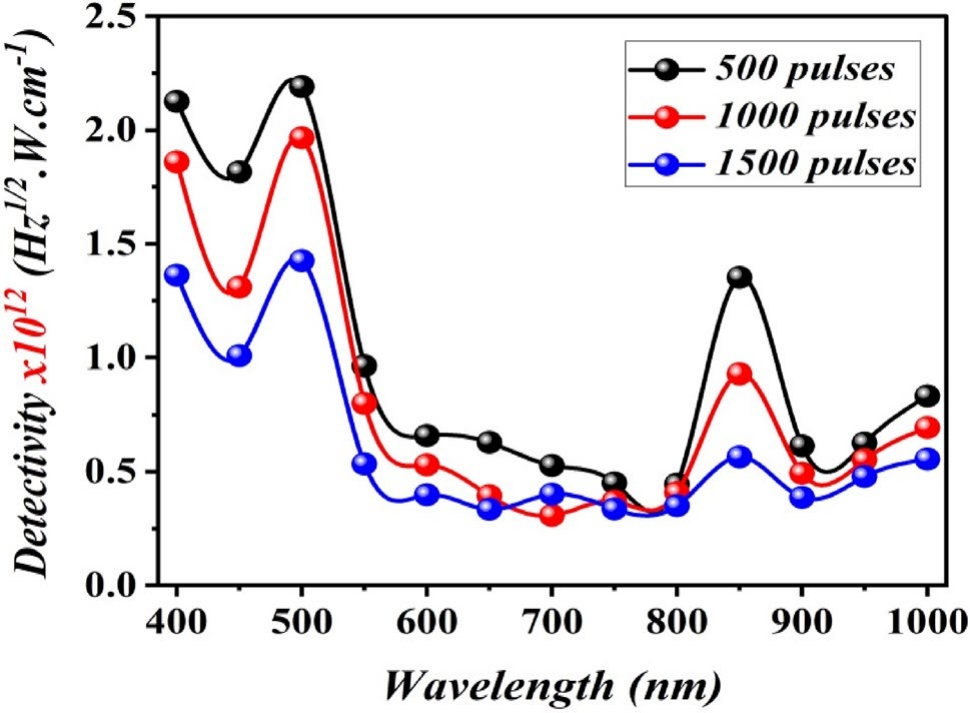
Effect of laser pulses on specific detectivity for B_4_C/p-Si photodetectors fabricated at different number of laser pulses.

The first peak was attributed to the absorption edge of B_4_C, which agrees with the value of the estimated optical energy gap, whereas the second peak is may probably due to the absorption edge of the silicon substrate^31^. The photodetector prepared at 1000 pulses has two response peaks located at 500 nm with responsivity of 0.6 A W^−1^ and at 850 nm with responsivity of 0.28 A W^−1^, and the photodetector prepared at 1500 pulses has responsivity of 0.43 A W^−1^ at 500 nm and 0.17 A W^−1^ at 850 nm. When the number of laser pulses increases, the responsivity decreases due to the effect of particles agglomeration and increases the electrical resistivity of B_4_C NPs^32^. The responsivity of the photodetectors at 850 nm was lower than that at 500 nm because the wavelengths shorter than 550 nm were absorbed at B_4_C film and the light with wavelengths longer than 550 nm was transmitted from B_4_C film to the silicon substrate and produced e–h pairs, which contributed to increasing the responsivity in the visible and near infrared regions. Figure 16 displays the specific detectivity of B_4_C/p-Si heterojunction photodetectors, which was measured from the following equation:

$${D}^{*}=\frac{{R}_{\lambda }{\left(A\right)}^\frac{1}{2}}{\sqrt{2e}{I}_{d}} $$

where A is the sensitive area of the photodetector and I_d_ is the dark current of the photodetector.

The photodetector prepared with 500 pulses has a detectivity of 2.18 × 10^12^ Jones at 500 nm, which is larger than that of the photodetectors fabricated with 1000 and 1500 pulses. This can be explained by increasing the laser pulses, which reduces detectivity by increasing the photodetector's noise current. Table 3 lists the figures of merit of the photodetectors as a function of the number of laser pulses at a bias voltage of − 5 V. The noise equivalent power NEP was calculated from detectivity. A smaller NEP means that the photodetector has a better ability to detect weak signals.

**Table 3:** Figures of merit of the photodetectors fabricated with different laser pulses at 500 nm.

No. laser pulses	R_λ_ (A W^−1^)	D*(Jones) × 10^12^	EQE (%)	NEP (pW)
500	0.66 at 500 nm	2.18	164	0.45
1000	0.6 at 500 nm	1.96	147	0.68
1500	0.43 at 500 nm	1.42	106	0.94

**Figure 17:**
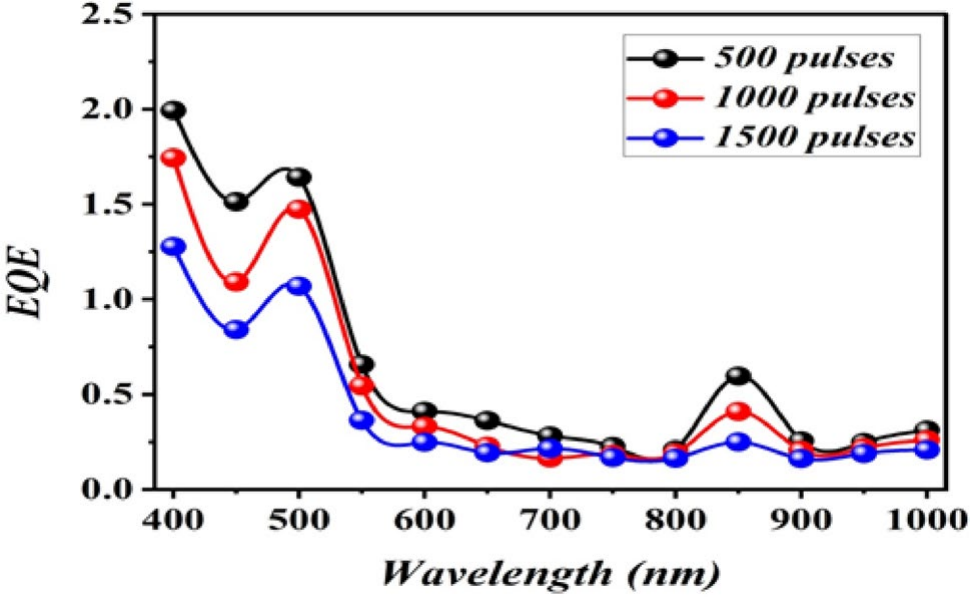
EQE as a function of wavelength of B_4_C/p-Si photodetectors fabricated at different number of laser pulses at bias voltage of 5 V.

Figure 17 shows the external quantum efficiency EQE of photodetectors prepared with varying numbers of laser pulses. The quantum efficiency is defined as the ratio of the number of generated electrons collected by the photodetector to the number of incident photons^33^ and it can be calculated from the following equation:

$$EQE=\frac{1240 \,{R}_{\lambda }}{\lambda\, (nm)} $$

The maximum EQE was 2.18 at 500 nm for the photodetector fabricated with 500 pulses. The EQE was higher than unity due to the widening of the depletion layer width as a results of increasing the reverse bias voltage. This result suggesting that the photodetector is fully depleted at this reverse voltage, resulting in efficient photogenerated carrier collection. Additionally, via promoting charge injections and generating free carriers, the applied bias voltage improves quantum efficiency significantly by absorbing photons and causing the photomultiplication effect.

**Figure 18:**
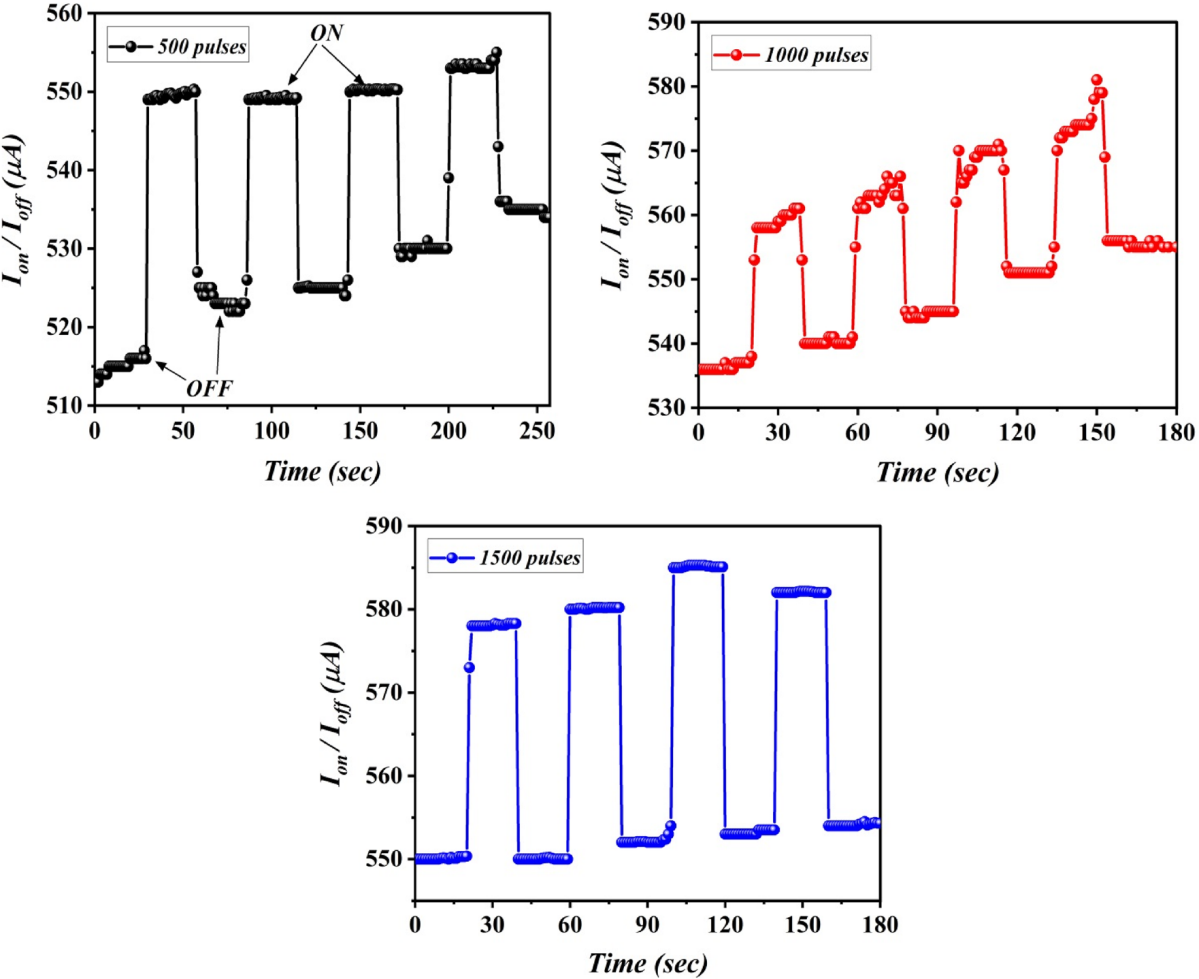
I–t curves of B_4_C/p-Si photodetectors fabricated at different number of laser pulses with response/recovery time.

As shown in Fig. 18, an ON/OFF measurement was performed to investigate the response/recovery time and switch behavior of the constructed photodetectors. These measurements were taken over a 20-s period with a light power of 35 mW cm^−2^ and various ON/OFF states. As illustrated in Fig. 18, the resulting current generally grows swiftly to a specified value as laser pulses increase. The photo-responsive behavior was discovered to be co-dependent on the laser pulses used. The photodetector's photocurrent value increased quickly to its maximum at 1.1 s response time and then decreased to its preliminary value at 1 s recovery time, for heterojunctions fabricated with 500 pulses. The photodetector constructed with 1000 pulses shows that its photocurrent maximum increased at a response time of 1.4 s and slowly reduced to its preliminary value (I_d_) with a decay time of 2 s. While the photodetector was fabricated with 1500 pulses, its photocurrent reached its maximum value at a response time of 1.5 s and subsequently declined to its minimum value (dark current) at a decay time of 0.8 s.

**Figure 19:**
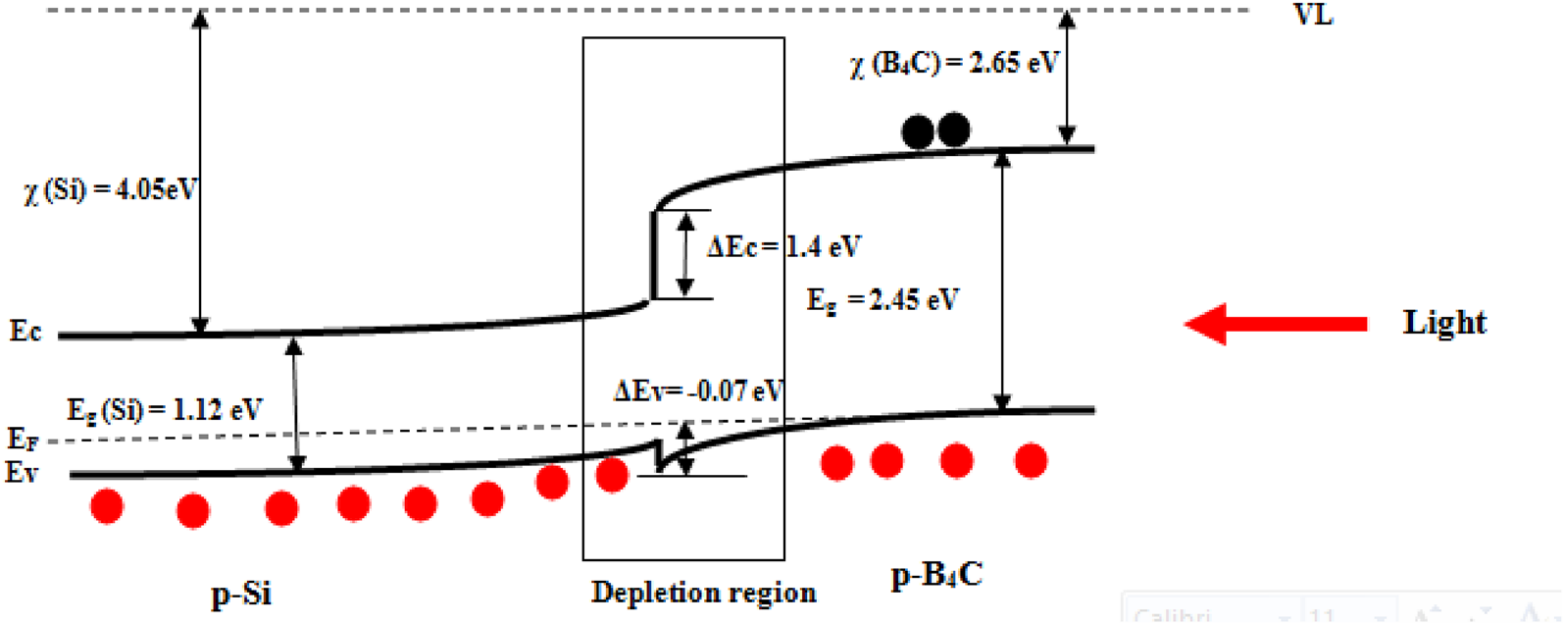
Illuminated energy band diagram of p-B_4_C/p-Si heterojunction photodetector prepared at 500 laser pulses.

The energy band diagram of the p-B_4_C/p-Si heterojunction photodetector under illumination and reveres bias is shown in Fig. 19. The conduction band offset was calculated as ΔEc = χ_Si_ − χ _B4C_ = 4.05–2.65 = 1.4 eV and valence band offset ΔEv = (E_g B_4_C_ − E_g Si_) − (χ_Si_ − χ _B4C_) =  − 0.07 eV. The photogenerated holes drifted from silicon to B_4_C due to the small value of ΔEv resulting in an increase in the photocurrent of the photodetector.

## Conclusions

For the first time, laser ablation of B_4_C nanoparticles in liquid was demonstrated. The effect of the number of laser pulses on the morphological, optical, and electrical properties of B_4_C nanoparticles was examined and analyzed. The XRD results confirmed that the synthesized B_4_C nanoparticles were polycrystalline in nature with a rhombohedral structure, and the crystallinity of the nanoparticles improved as the laser pulses increased. As laser pulses increase, UV–Vis absorbance spectra slightly increase, and the energy gap decreases from 2.45 to 2.38 eV. The results of PL showed that photodetectors fabricated at 500, 1000, and 1500 pulses exhibit single emission peaks centered at 485, 495, and 500 nm, respectively, which matched with UV–Vis results. The synthesized B_4_C nanoparticles have spherical particles with an average size that depends on the number of laser pulses. The Raman spectra of B_4_C nanoparticles exhibit six vibration modes with intensities dependent on the number of laser pulses. A high-performance B_4_C/p-Si heterojunction photodetector was designed, fabricated, and characterized. The photodetectors have a spectral operating region of 400–1000 nm. A photodetector designed with 500 pulses showed the maximum responsivity was 0.66 A W^−1^ at 500 nm. A maximum detectivity and quantum efficiency of 1.64 × 10^2^%, and 2.18 × 10^12^ Jones, respectively, were reached for a photodetector fabricated at 500 laser pulses. Our study shows a simple and low-cost technique for constructing high-performance photodetectors working in the visible and near-infrared regions can be utilized to detect weak power signals. Based on the switching analysis, the photodetectors have a typical response/decay time, which is suitable for optical communication applications.

## Data Availability

The datasets generated and/or analyzed during the current study are available from the corresponding author (R.A.I) on reasonable request.
